# Supplementary material for: Emotional Intelligence and Burnout in Healthcare Professionals: A Hospital-Based Study
Source: Healthcare (Basel). 2025 Jul 29;13(15):1840. doi: 10.3390/healthcare13151840 (PMC12346813; doi:10.3390/healthcare13151840)
Supplement: Supplementary file 1 [file healthcare-13-01840-s001.zip › healthcare-3643745-supplementary.pdf]

Table S1: The Trait Emotional Intelligence Questionnaire-Short Form (TEIQue-SF)

Instructions: Please answer each statement below by putting a circle around the number that best reflects your degree of agreement or disagreement with that statement. Do not think too long about the exact meaning of the statements. Work quickly and try to answer as accurately as possible. There are no right or wrong answers. There are seven possible responses to each statement ranging from 'Completely Disagree' (number 1) to 'Completely Agree' (number 7).

1 . . . . 2 . . . . 3 . . . . 4 . . . . 5 . . . . 6 7

Completely Disagree ... **Completely Agree**

1. Expressing my emotions with words is not a problem for me.	1	2	3	4	5	6	7
2. I often find it difficult to see things from another person's viewpoint.	1	2	3	4	5	6	7
3. On the whole, I'm a highly motivated person.	1	2	3	4	5	6	7
4. I usually find it difficult to regulate my emotions.	1	2	3	4	5	6	7
5. I generally don't find life enjoyable.	1	2	3	4	5	6	7
6. I can deal effectively with people.	1	2	3	4	5	6	7
7. I tend to change my mind frequently.	1	2	3	4	5	6	7
8. Many times, I can't figure out what emotion I'm feeling.	1	2	3	4	5	6	7
9. I feel that I have a number of good qualities.	1	2	3	4	5	6	7
10. I often find it difficult to stand up for my rights.	1	2	3	4	5	6	7
11. I'm usually able to influence the way other people feel.	1	2	3	4	5	6	7
12. On the whole, I have a gloomy perspective on most things.	1	2	3	4	5	6	7
13. Those close to me often complain that I don't treat them right.	1	2	3	4	5	6	7
14. I often find it difficult to adjust my life according to the circumstances.	1	2	3	4	5	6	7
15. On the whole, I'm able to deal with stress.	1	2	3	4	5	6	7
16. I often find it difficult to show my affection to those close to me.	1	2	3	4	5	6	7
17. I'm normally able to "get into someone's shoes" and experience their emotions.	1	2	3	4	5	6	7
18. I normally find it difficult to keep myself motivated.	1	2	3	4	5	6	7
19. I'm usually able to find ways to control my emotions when I want to.	1	2	3	4	5	6	7
20. On the whole, I'm pleased with my life.	1	2	3	4	5	6	7
21. I would describe myself as a good negotiator.	1	2	3	4	5	6	7
22. I tend to get involved in things I later wish I could get out of.	1	2	3	4	5	6	7
23. I often pause and think about my feelings.	1	2	3	4	5	6	7
24. I believe I'm full of personal strengths.	1	2	3	4	5	6	7
25. I tend to "back down" even if I know I'm right.	1	2	3	4	5	6	7
26. I don't seem to have any power at all over other people's feelings.	1	2	3	4	5	6	7
27. I generally believe that things will work out fine in my life.	1	2	3	4	5	6	7
28. I find it difficult to bond well even with those close to me.	1	2	3	4	5	6	7
29. Generally, I'm able to adapt to new environments.	1	2	3	4	5	6	7
30. Others admire me for being relaxed.	1	2	3	4	5	6	7

Trait Emotional Intelligence Questionnaire – Short Form (TEIQue-SF). This 30-item form includes two items from each of the 15 facets of the TEIQue. Items were selected primarily on the basis of their correlations with the corresponding total facet scores, which ensured broad coverage of the sampling domain of the construct. The –SF can be used in research designs with limited experimental time or wherein trait EI is a peripheral variable. Although it is possible to derive from it scores on the four trait EI factors, in addition to the global score, these tend to have somewhat lower internal consistencies than in the full form of the inventory. The –SF does not yield scores on the 15 trait EI facets.

Scoring information for the TEIQue-SF is available at: http://www.psychometriclab.com/Home/Default/14 Please note that we cannot provide any advice on how to run the syntax in SPSS or other statistical software.

Please make sure you read the FAQ section at http://www.psychometriclab.com/Home/Default/18. In particular, note that we do not provide free information regarding norms or free feedback reports. Norms and reports are available for a fee (email admin@teique.com for quotes).

Reference for the TEIQue-SF: Petrides, K. V. (2009). Psychometric properties of the Trait Emotional Intelligence Questionnaire. In C. Stough, D. H. Saklofske, and J. D. Parker, *Advances in the assessment of emotional intelligence*. New York: Springer. DOI: 10.1007/978-0-387-88370-0_5

For more information about the trait emotional intelligence research program go to: www.psychometriclab.com

Please note that any and all commercial use of this instrument, or any adapted, modified, or derivative works thereof, is strictly prohibited.

Table S2: Burnout syndrome assessment tool

Instructions

For each question, mark the in the column that most applies. Then click the 'Calculate Total' button to add up your score and check your result using the scoring table underneath.

Statements	Not at All	Rarely	Sometimes	Often	Very Often
I feel run down and drained of physical or emotional energy.					
2I have negative thoughts about my job.					
3I am harder and less sympathetic with people than perhaps they deserve.					
4I am easily irritated by small problems, or by my co-workers and team.					
5I feel misunderstood or unappreciated by my co-workers.					
6I feel that I have no one to talk to.					
7I feel that I am achieving less than I should.					
8I feel under an unpleasant level of pressure to succeed.					
9I feel that I am not getting what I want out of my job.					
10I feel that I am in the wrong organization or the wrong profession.					
11I am frustrated with parts of my job.					
12I feel that organizational politics or bureaucracy frustrate my ability to do a good job.					
13I feel that there is more work to do than I practically have the ability to do.					
14I feel that I do not have time to do many of the things that are important to doing a good quality job.					
15I find that I do not have time to plan as much as I would like to.					

Not at All - 1

Rarely - 2

Sometimes - 3

Often - 4

Very Often - 5

Score Interpretation

- 15-18 No sign of burnout here.
- 19-32 Little sign of burnout here, unless some factors are particularly severe.
- 33-49 Be careful – you may be at risk of burnout, particularly if several scores are high.
- 50-59 You are at severe risk of burnout – do something about this urgently.
- 60-75 You are at very severe risk of burnout – do something about this urgently
